# Risk factors of gallbladder cancer in Karachi-a case-control study

**DOI:** 10.1186/1477-7819-9-164

**Published:** 2011-12-09

**Authors:** A Rehman Alvi, Nadeem Ahmed Siddiqui, Hasnain Zafar

**Affiliations:** 1 Department of Surgery, Aga Khan University Hospital, Karachi-74800, Pakistan

**Keywords:** Gall bladder cancer, Prophylactic cholecystectomy, Risk factors for gallbladder cancer

## Abstract

**Background:**

Gallbladder carcinoma (GC) is a relatively rare malignancy worldwide but is the second commonest gastrointestinal cancer in Pakistani women. Gallstones have a positive association with GC but other factors also influence in causation.

**Methods:**

This is a retrospective case control study over a period of 19 years. The cases (Group A) were patients with histopathological proven carcinoma gallbladder (N = 60) and controls were patients with cholelithiasis but no carcinoma gallbladder on histopathology (N = 120). Multivariate regression analysis was done to calculate the odds ratio, 95% confidence interval and P-Value. A positive relationship was found between size of stone > 1 cm, solitary stone, age > 55 years and multi-parity in women.

**Results:**

There were 60 patients in Group A and 120 patients in Group B. mean age of diagnosis in Group A patients was 57 ± 2.4 years while mean age of diagnosis in Group B patients was 48 ± 1.35 years. Sixty seven percent of cancer group patients were female as compared to 78% females in non-cancer group. In Group A, 69% of female patients were multiparous (parity of more than 5) while 43% of group B patients were multiparous. For body mass index (BMI), both groups were not very different in our study population i.e. around 78% patients in each group has BMI of more than 23 Kg/m2. In Group A, 37% (n = 22) have solitary stones as compared to 15% (n = 18) in group B. similarly Group A patients has larger stone size as compared to Group B i.e.59% (n = 36) patients in Group A have stones of more than 1 cm when compared to 35% (n = 41) patients in Group B. After using multivariate regression analysis, age more than 55 years (OR - 7.27, p value- < 0.001), solitary stone (OR - 3.33, p value - 0.002) and stone of more than 1 cm (OR - 2.73, p value - 0.004) were found to be independent risk factors for development of gallbladder cancer.

**Conclusion:**

Most of the patients (78%) with GC were female, and the statistically significant risk factors were older age, solitary stones and stones size more than one centimeter. A case can be made for prophylactic cholecystectomy in such a high risk group. However a population based study is required to calculate the true incidence of GC in Karachi and a prospective multi center study is needed to produce strong evidence for screening and prophylactic cholecystectomy.

**Trial Registration:**

As this was a retrospective review of medical records, as per institution policy, its gives waiver from any registration (ethical/trial).

## Background

Gallbladder carcinoma (GC) was first described by Maximilian Stoll in 1777 and more than 200 years later it is still considered to be a highly malignant disease with a poor survival rate [1-3]. The clinical presentation of GC is non- specific. It is often recognized late with the diagnosis being established during advanced stages of disease. Survival is less than 5 years survival in 90% of cases [2,3].

The incidence of GC in any population varies widely among various geographic regions and ethnic groups ranging from 1 to 23 per 100,000 [2-7]. There are reported rising incidence of GC from Northern India and Southern Pakistan over the past two decades [3-5]. GC is the second commonest malignancy of gastrointestinal origin in Pakistani women. It is the most common cause of gastrointestinal cancer related mortality in females in the region [3,4].

While the etiology of GC remains obscure, it seems that more than one factor plays a role in the pathogenesis. A strong association is observed between gallstones and GC, suggesting that it is the most important risk factor. In addition genetic factors, diet, parity, obesity, bacterial infection, poverty, benign neoplasm of gallbladder, congenital abnormalities and porcelain gallbladder are also postulated to the pathogenesis of GC [5-11]^.^

The aim of this case-control study was to identify the risk factors of GC, comparing patients with cholelithiasis and gallbladder cancer patients to patients with cholelithiasis but no cancer on histopathology.

## Methods

It was a retrospective case control study conducted at Aga Khan University Hospital, Karachi, Pakistan. In this review, we included patients with cholelithiasis and gall bladder cancer and patients with cholelithiasis without gall bladder cancer over period of 19 years i.e. 1988 to 2007.

### Cases and control selection

All the patients with cholelithiasis and histologically proven gallbladder cancer were retrieved through hospital's electronic data base system using ICD-9 coding system. There were 60 patients in this group and were selected as CASES for the study (Group A). For these cases, CONTROLS were selected of those patients who have gall stones without gall bladder cancer on histology (Group B). Total of 120 patients were selected as controls in Group B (case: control - 1:2). Group B controls were selected through computer generated software, which had randomly selected seven patients from every year (1988-2007), who underwent cholecystectomy for cholelithiasis (total 133 patients for 19 years). After reviewing these 133 patient's records, 13 were excluded from study due to selected exclusion criteria, so 120 patients were included in the study as controls.

### Inclusion and exclusion criteria

In Group A, all adult patients of age 18-75 years with gall bladder cancer long with gall stones were included in the study. Patients with gallbladder cancer without gallstones, missing and/or incomplete records, and patients with other concomitant malignancy were excluded from study.

In Group B, all adult patients (18-75 years), who underwent laparoscopic cholecystectomy for gall stone disease, were included in the study. Patients who underwent laparoscopic cholecystectomy without gallstones (polyp, biliary dyskinesia), any history of previous malignancy and those with incomplete and/or missing record were excluded from the study.

### Data collection

After selecting cases and control groups, patient's medical records were reviewed retrospectively and information was recorded on predefined Performa. This Performa included all basic demographic details, clinical spectrum and information regarding documented risk factors for gallbladder cancer (parity, body mass index, alcohol, smoking, family history, oral contraceptive pill use, typhoid carrier state, stone number and size etc.).

As this was a retrospective review of medical records without any intervention, hospital ethical review board approval was not taken.

Both the groups were compared for following risk factors, age of diagnosis (age more than 55 being risk), BMI (BMI of more than 23 kg/m^2 ^being risk), parity (multiparity being risk factor), number of stones (solitary stones being risk factor) and size of stones (size more than 1 cm being risk). Number and size of stones were recorded from initial ultrasound report. As in our institution, stone numbers are being reported as either single or multiple, so actual number of stones was not available to calculate mean or median number of stone. For stone size, size of largest stone documented on preoperative ultrasound was used.

### Statistical analysis

Data was entered and analyzed using SPSS version 14. Age was calculated in means and medians. Frequency tables were used to compare basic demographic, clinical and other desired characteristics. Comparison of characteristics between two groups was done using chi square test. Factors which came out to be different with statistical significance, multivariate regression analysis was used to calculate Odd ratio (OR), confidence interval (CI) and p values. CI of 95% and p value of less than 0.05 was considered statistically significant.

## Results

There were 60 patients in Group A and 120 patients in Group B. mean age of diagnosis in Group A patients was 57 ± 2.4 years while mean age of diagnosis in Group B patients was 48 ± 1.35 years. Sixty seven percent of cancer group patients were female as compared to 78% females in non cancer group. In Group A, 69% of female patients were multiparous (parity of more than 5) while 43% of group B patients were multiparous. For BMI, both groups were not very different in our study population i.e. around 78% patients in each group has BMI of more than 23 Kg/m^2^. Other documented risk factors of gallbladder cancer like typhoid carrier state, oral contraceptive pill use, smoking, alcohol, family history were also tried to analyze, but for these risk factors, data was either insufficient or difference between two groups was too small. Basic demographic, clinical and risk factor details of two groups are given in table 1.

**Table 1:** showing comparison of basic demographic, clinical and ultrasonographic details of two population

Variable	Cases n = 60 (%)	Controls n = 120 (%)	P-value
*Gender*			
Male	20(33.33)	26(21.6)	0.104
Female	40(66.66)	94(78.1)	

Age more than 55 years	42(68)	29(24)	**0.0001**

BMI more than 23 Kg/m^2^	46(77)	94(78)	0.78

Parity of more than 5	41(69)	51(43)	**0.005**

History of Hypertension	16(26)	28(23.3)	0.826

History of Diabetes	13(21.6)	17(14.1)	0.726

History of typhoid	nil	1	0.000*

Oral contraceptive pills use	nil	18)15)	0.000*

History of smoking	8(13.3)	4(3)	0.123

History of alcohol	2(3)	9(7)	0.000*

*Stone characteristics*			
Solitary stones	22(37)	18(15)	**0.005**
Size more than 1 cm	36(59)	41(35)	**0.015**
Mean stone size in cm	1.9	0.6	
Median of stone size in cm	2.1	0.8	

P-value calculated using chi-square test.* Unreliable significance values as this value is confounded by significant missing/small data.

Preoperative ultrasound findings were used to collect information regarding stone characteristics i.e. number and size of stones. In Group A, 37% (n = 22) have solitary stones as compared to 15% (n = 18) in group B. similarly Group A patients has larger stone size as compared to Group B i.e.59% (n = 36) patients in Group A have stones of more than 1 cm when compared to 35% (n = 41) patients in Group B. Mean stone size in Group A patients was 2.2 cm as compared to group B patients, in which mean stone size was 0.8 cm.

After comparing both groups for basic demographic characteristics and risk factors for gallbladder cancer, difference between two groups were calculated using chi square test (table 1). After initial comparison, age, BMI, parity, stone size and number of stones showed significant and/or marginal insignificant values. So these variables were included in multivariate regression analysis. Odd ratio (OR), confidence interval (CI) and p values obtained after multivariate analysis are shown in table 2.

**Table 2:** Multivariate regression analysis showing risk factors for gallbladder cancer

Variable	Cases n = 60(%)	Controls N = 120 (%)	Odds ratio (95% CI)	p-value
*Age in years*				
< 55	18(32)	91(76)	1	**< 0.0001**
> 55	42(68)	29(24)	7.27(3.66-14.45)	

*Parity*				
< 5	19(31)	69(57)	1	0.064
> 5	41(69)	51(43)	6.99(0.89-54.84)	

*BMI in Kg/m^2^*				
< 23	14(23)	26(22)	1	0.244
> 23	46(77)	94(78)	1.98(0.62-6.28)	

*Stone size in cm*				
< 1	24(41)	79(65)	1	**0.004**
> 1	36(59)	41(35)	2.73(1.37-5.4)	

*Number of stones*				
Multiple	38(63)	102(85)	1	**0.002**
single	22(37)	18(15)	3.33(1.57-7.08)	

CI - confidence interval

BMI - body mass index

After using multivariate regression analysis, age more than 55 years (OR - 7.27, p value- < 0.0001), solitary stone (OR - 3.33, p value - 0.002) and stone of more than 1 cm (OR - 2.73, p value - 0.004) were found to be independent risk factors for development of gallbladder cancer.

## Discussion

Gallbladder carcinoma is a relatively rare neoplasm but shows a marked geographic, ethnic and socioeconomic variation. There are significant changing trends in the incidence of gallbladder carcinoma over the last three decades with decrease in incidence in developed and increase in incidence in developing countries [1-3].

The highest incidence of gallbladder carcinoma is reported more recently from the Indian-Subcontinent including India and Pakistan (18-23/100,000) [2]. These differences can have several interpretations, but they refer to the worldwide distribution of gall stones which are the most important risk factor for GC. Although a small proportion of patients (1-3%) with gall stones developed GC. But at the same time there is an inverse relationship between the incidence of GC and rate of cholecystectomy. In many European countries and USA there is significant increase in cholecystectomy rate and at the same time lower incidence of GC [5-7].

Ethnic, family predisposition and geographic variation suggests that besides gall stones other factors also contributes to the occurrence of GC e.g. genetic predisposition, shared metabolic and life style factors including obesity, dietary habits, infection and parity [12-16].

This study suggests that the major risk factor for GC is gall stones, which were present in 100% of cases and there are high reported risk of GC with stones (odds ratio 19-23) [2,3]. In this study statistically significant differences between cases and control were identified as size of stone > 1 cm (odd ratio 2.73 and P-Value = 0.004) and solitary stone (odds ratio 3.33 and P-Value = 0.002) these factors have been reported in other studies to be major risk factors of GC along with volume of stones [8,17]. In the Indian-subcontinent gallstones formation occurs at a younger age as compared to the Western population and this association has been described in patients who have gall stones for more the twenty years prior to GC [1-5]. The theoretical basis for this phenomenon is that of inflammation, trauma and infection may predispose the patient to epithelial dysplasia and adenocarcinoma formation. This may be the reason that large and high volume stones have more impact on the risk of GC, possibly because of greater duration and intensity of epithelial irritation [8,17-19].

The other risk factor of GC was age greater than 55 years (odds ration 7.27 and P-Value = 0.0001) with the mean age of such cases at 58.3 year compared to controls at 44.6 years with peak incidence being in the sixth decade of life. Majority of the patients (more than 89%) were less the 60 years of age. GC develops at a young age in Indian and Pakistani populations because of gall stones developing at a younger age [2,5,6]. The age difference may be over-estimated as the life expectancy in Pakistan is low (male 62 and female 65 years). However a study from Pakistan has reported 80% GC in patients less than 60 years of age after adjusting age and sex distribution [3]. In western population the majority of GC (more than 80%) develops in older than 60 years [1,2].

Parity showed a strong correlation with GC in this study population (odds ratio 6.99 and P-Value = 0.002) and increase risk of GC when parity was more than five. The positive correlation of parity with GC has been reported worldwide with odds ratio of 6.15 to 21.3 and this has been attributed to menarche at a younger age, early age at first pregnancy, multiple pregnancies and prolonged period of fertility. All these factors may increase the risk of GC secondary to elevated levels of estrogen and progesterone. However oral contraceptive use is not associated with a higher frequency of GC.

The wide distribution between geographic and ethnic groups also indicate a role for genetic predisposition and life style variations, including dietary habits, smoking, chewing tobacco and use of other addictive substances as has been reported [1-3,6,20]. GC risk has been positively associated with low total caloric intake, low fiber intake in Pakistan (0dds ratio 4-6.9). Another risk factor was smoking tobacco (odds ratio 2.7) and was common in low socioeconomic class with defective diet and dietary habits [3]. Studies from India where GC has the highest incidence have also reported positive correlation of life style with GC and suggested high carbohydrate diet, low protein and low fiber diet in a low socioeconomic class have strong relation with gallstone and GC (odds ratio 1.4-30) [6,20,21]. The studies from Western countries and South America have also reported positive relation between GC and life style changes [13].

Familial occurrence of gallstones has been reported in several studies but only few have assessed a family history of gallstones in GC etiology. One of the population based study reported a 57-fold increase in GC with gall stones and positive family history of gallstones [12]and a national epidemiological study from Sweden has reported a high risk for gallbladder cancer (odds ratio 5.21) with family history of gall stones.

A disadvantage of the present study is like that of any retrospective studies where there were missing or incomplete information available. The information about other risk factors like family history of gallstone, life style information, fertility and typhoid career state were either insufficient or incomplete for statistical analysis.

## Conclusion

This study found statistically significance positive correlation of gallbladder carcinoma with large sized and solitary gall stones. Multi-parity and increase age seems to play an important role in causation of GC. Based on the results of the present study, a case can be made for prophylactic cholecystectomy as a preventive strategy in a high risk group of patients with asymptomatic gallstones. Early elective cholecystectomy for symptomatic gallstones may reduce the chances of gallbladder carcinoma in countries where there is reported high incidence of GC including South of Pakistan (Karachi). However a population based study is required to calculate the true incidence of GC in Karachi and a multi-center study is needed to produce strong evidence for screening and prophylactic cholecystectomy in high risk patients.

## List of Abbreviations

GC: Gallbladder cancer; BMI: Body mass index.

## Competing interests

The authors declare that they have no competing interests.

## Authors' contributions

AR: Contributed in concept, study design, data analysis, result interpretation and manuscript writing.

NAS: Participated in study conceptualization and conduct, data collection, data analysis, drafting manuscript and proof reading.

HZ: Contributed in conduct of study, data collection, data interpretation and proof reading.

All authors read and approved the final manuscript
